# Rapid Increase in Lymphogranuloma Venereum among HIV-Negative Men Who Have Sex with Men, England, 2019

**DOI:** 10.3201/eid2710.210309

**Published:** 2021-10

**Authors:** Mateo Prochazka, Hannah Charles, Hester Allen, Michelle Cole, Gwenda Hughes, Katy Sinka

**Affiliations:** Public Health England, London, UK

**Keywords:** Lymphogranuloma venereum, LGV, *Chlamydia trachomatis*, men who have sex with men, HIV/AIDS, epidemiology, infectious disease, sexually transmitted infections, England

## Abstract

Incidence of lymphogranuloma venereum increased in England during 2018–2019, after a period of decline. Our retrospective analysis of national surveillance data identified a rapid increase in diagnoses among HIV-negative men who have sex with men. These findings indicate a need for sustained surveillance and targeted public health action.

Lymphogranuloma venereum (LGV) is an invasive form of *Chlamydia trachomatis* infection. In high-income countries, LGV is concentrated among gay, bisexual, and other men who have sex with men (MSM) (*1*). Although LGM was initially characterized as affecting predominantly MSM living with HIV who have symptomatic proctitis (*2*,*3*), recent evidence suggests considerable underestimation of the actual extent of LGV among MSM in Europe because of insufficient testing of asymptomatic persons (*4*). Changes to UK guidelines (*5*) and testing practices of several large London clinics have led to expanded testing in MSM regardless of HIV status, leading to increased diagnoses among HIV-negative MSM and those without symptoms of proctitis (*6*–*9*). Expanded testing may have precipitated a decline in incidence during 2016–2018 (*9*); however, 2019 saw the highest number of LGV diagnoses reported in England since routine testing began in 2004, and test positivity increased from 8.2% in 2018 to 9.0% in 2019 (*10*). In this study, we investigated the changing epidemiology of LGV among MSM in England during 2015–2019 and risk factors associated with recent cases.

## The Study

We conducted a retrospective analysis of adult (>16 years of age) MSM in England who visited sexual health service (SHS) sites during January 1, 2015–December 31, 2019. We obtained data from the Genitourinary Medicine Clinic Activity Dataset Sexually Transmitted Infection (STI) Surveillance System, which has recorded LGV diagnoses, obtained through multiplex reverse transcription PCR (*4*), since 2011. We included all SHS site visits by men who self-reported as MSM in England during 2015–2019. We cleaned and deduplicated data according to a routine practice described previously (*11*). We described the number of annual LGV diagnoses during 2015–2019 by age group, race (White [e.g., White British, White Irish, or White other background] and non-White), place of residence (London or rest of England), region of birth (United Kingdom, Europe, Asia, Oceania, Latin America and the Caribbean [LAC], North America, and Africa), history of a bacterial STI in the previous year (defined as having a recorded diagnoses of chlamydia, gonorrhea, or syphilis in the 365 days before attendance), and HIV status at time of LGV diagnosis. We used quarterly data on LGV diagnoses stratified by HIV status to examine changes over time in the proportion of diagnoses that were made among MSM who are HIV-negative or with unknown HIV status. We used generalized linear models with logarithmic function, Poisson distribution, and robust variances to identify the changes in risk for LGV in 2019 by quarter. We included covariates in the model if they showed strong association with an LGV diagnosis in the bivariate analyses (p<0.05). We adjusted the final model for HIV status, history of a previous bacterial STI, region of birth, and age group. We performed all data analyses using Stata 15.1 (https://www.stata.com).

Public Health England collects pseudonymized, electronic data on all STI tests and diagnoses from all commissioned SHS sites in England (*11*). Public Health England has approval to handle data obtained by the Genitourinary Medicine Clinic Activity Dataset STI Surveillance System under Regulation 3 of the Health Service (Control of Patient Information) Regulations 2002 (https://www.gov.uk/government/publications/hiv-and-sti-data-sharing-policy).

**Figure 1:**
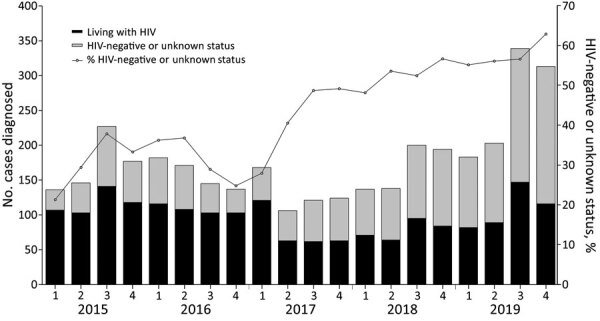
Annual and quarterly number of lymphogranuloma venereum diagnoses among men who have sex with men, by HIV status, England, 2015–2019.

Of 2,116,345 SHS visits by MSM during 2015–2019, we identified 3,461 diagnoses of LGV (Table 1). The highest number of LGV diagnoses was recorded in 2019 (n = 1,018); this increase was mainly attributed to increases in diagnoses in the third and fourth quarter of the year (Figure 1). The proportion of diagnoses among MSM who are HIV-negative or with unknown HIV status increased from 31.4% in 2015 to 58.4% in 2019 (Table 1). In 2019, most LGV diagnoses in MSM were among White MSM (72.7%) and MSM residing in London (79.1%). A total of 614 (60.3%) LGV diagnoses in 2019 were made among those who had a bacterial STI diagnosis in the previous year (Table 1).

**Table 1:** Characteristics of men who have sex with men who had lymphogranuloma venereum diagnosed, England, 2015–2019*

Characteristic	No. (%) patients				
	2015	2016	2017	2018	2019
Total no. patients	663	620	505	655	1,018
Age group, y					
16–24	45 (6.8)	31 (5.0)	40 (7.9)	27 (4.1)	81 (8.0)
25–34	250 (37.7)	234 (37.7)	166 (32.9)	243 (37.1)	377 (37.0)
35–44	229 (34.5)	208 (33.6)	141 (27.9)	219 (33.4)	301 (29.6)
45–54	103 (15.5)	115 (18.6)	124 (24.6)	122 (18.6)	180 (17.7)
55–64	26 (3.9)	24 (3.9)	25 (5.0)	32 (4.9)	68 (6.7)
*>*65	4 (0.6)	5 (0.8)	5 (1.0)	11 (1.7)	7 (0.7)
Unknown	6 (0.9)	3 (0.5)	4 (0.8)	1 (0.2)	4 (0.4)
Residence					
London	512 (77.2)	469 (75.7)	362 (71.7)	507 (77.4)	806 (79.1)
Rest of England	151 (22.8)	151 (24.4)	143 (28.3)	148 (22.6)	212 (20.8)
Race					
White	499 (75.3)	479 (77.3)	383 (75.8)	485 (74.0)	740 (72.7)
Non-White	124 (18.7)	121 (19.5)	99 (19.6)	125 (19.1)	208 (20.4)
Unknown	40 (6.0)	20 (3.2)	23 (4.6)	45 (6.9)	70 (6.9)
Region of birth					
United Kingdom	333 (50.2)	317 (51.1)	248 (49.1)	300 (45.8)	443 (43.5)
Europe	159 (24.0)	139 (22.4)	112 (22.2)	172 (26.3)	256 (25.2)
Asia	38 (5.7)	33 (5.3)	25 (5.0)	38 (5.8)	50 (4.9)
Oceania	10 (1.5)	13 (2.1)	7 (1.4)	18 (2.8)	28 (2.8)
Latin America and Caribbean	51 (7.7)	41 (6.6)	45 (8.9)	44 (6.7)	111 (10.9)
Northern America	9 (1.4)	7 (1.1)	22 (4.4)	18 (2.8)	18 (1.8)
Africa	14 (2.1)	23 (3.7)	13 (2.6)	25 (3.8)	31 (3.1)
Unknown	49 (7.4)	47 (7.6)	33 (6.5)	40 (6.1)	81 (8.0)
Bacterial STI in previous year					
Yes	310 (46.8)	276 (44.5)	217 (43.0)	356 (54.4)	614 (60.3)
No	353 (53.2)	344 (55.5)	288 (57.0)	299 (45.7)	404 (39.7)
HIV status					
Living with HIV	455 (68.6)	420 (67.7)	299 (59.2)	302 (46.1)	424 (41.7)
Negative or unknown status	208 (31.4)	200 (32.7)	206 (40.8)	353 (53.9)	594 (58.4)

*STI, sexually transmitted infection.

**Figure 2:**
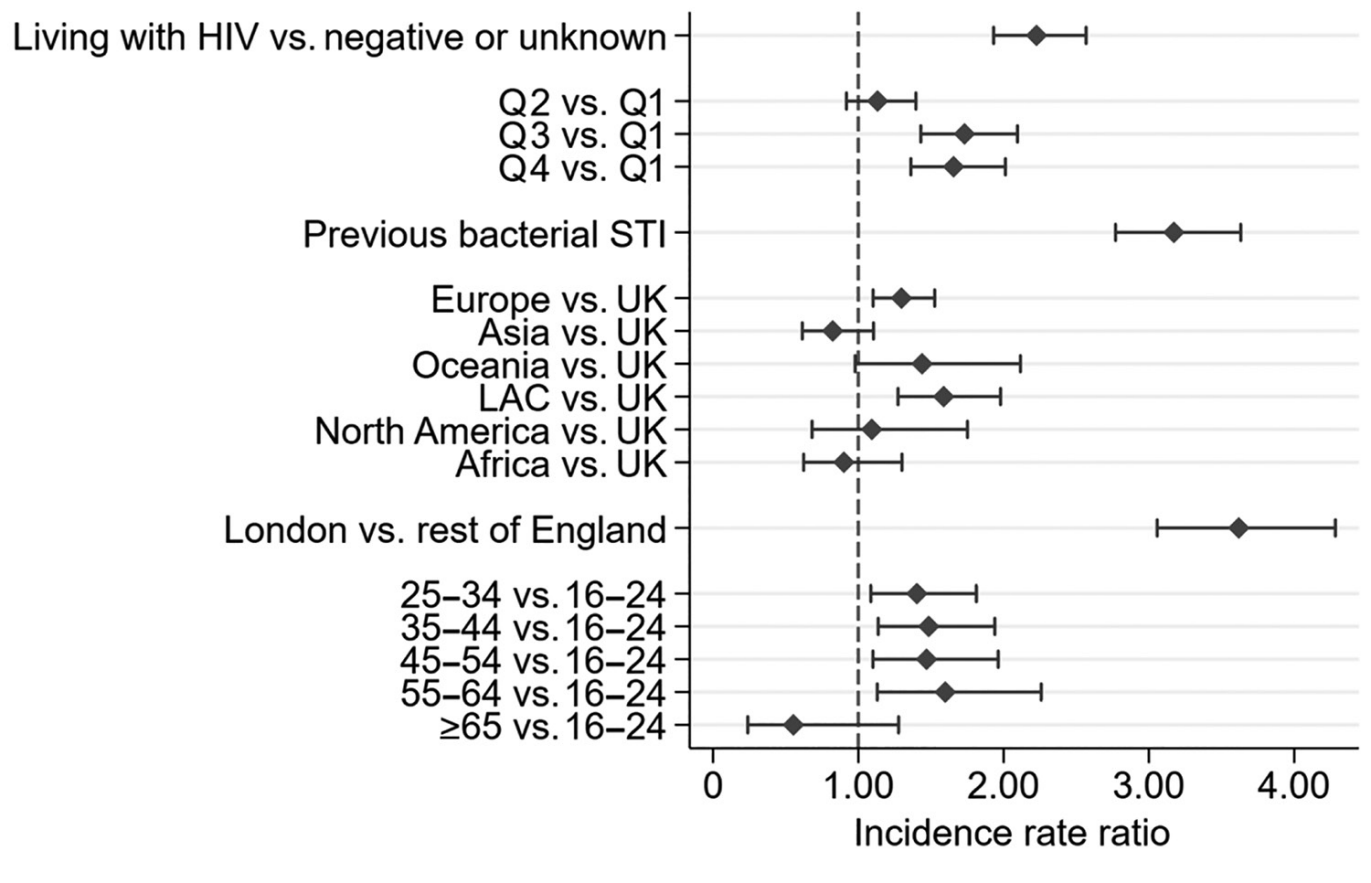
Adjusted incidence rate ratios for lymphogranuloma venereum among men who have sex with men, England, 2019. Diamonds indicate effect estimates (incidence rate ratio); error bars indicate 95% CIs for those estimates. LAC, Latin America and the Caribbean; Q, quarter; STI, sexually transmitted infection; UK, United Kingdom.

Our regression analyses included 526,102 visits and 1,018 LGV diagnoses among MSM in England in 2019 (Table 2). Compared with quarter 1 of 2019, the risk for having LGV diagnosed was 73% higher in quarter 3 and 66% higher in quarter 4. Living with HIV, residing in London, and having a previous bacterial STI were strongly associated with an LGV diagnosis in the crude and adjusted models (Table 2). Being born in LAC or in Europe outside of the UK were also associated with increased risk for LGV (Figure 2).

**Table 2:** Crude and adjusted incidence rate ratios for lymphogranuloma venereum among men who have sex with men, England, 2019*

Characteristic	Crude incidence rate ratio (95% CI)	Adjusted† incidence rate ratio (95% CI)
Year		
Quarter 1	Referent	Referent
Quarter 2	1.10 (0.90–1.35)	1.13 (0.92–1.40)
Quarter 3	1.68 (1.40–2.02)	1.73 (1.43–2.10)
Quarter 4	1.49 (1.24–1.80)	1.66 (1.36–2.01)
HIV		
Negative or unknown status	Referent	Referent
Living with HIV	2.55 (2.26–2.90)	2.23 (1.93–2.57)
Bacterial STI in previous year		
No	Referent	Referent
Yes	4.01 (3.54–4.55)	3.17 (2.77–3.63)
Residence		
Rest of England	Referent	Referent
London	4.28 (3.68–4.98)	3.62 (3.06–4.28)
Region of birth		
United Kingdom	Referent	Referent
Europe	2.42 (2.07–2.82)	1.30 (1.10–1.53)
Asia	1.27 (0.95–1.70)	0.82 (0.61–1.10)
Oceania	2.96 (2.02–4.33)	1.44 (0.98–2.12)
Latin America and Caribbean	3.78 (3.07–4.65)	1.59 (1.27–1.98)
North America	1.89 (1.18–3.02)	1.09 (0.68–1.75)
Africa	1.60 (1.11–2.30)	0.90 (0.62–1.30)
Age group, y		
16–24	Referent	Referent
25–34	2.16 (1.70–2.75)	1.40 (1.09–1.81)
35–44	2.67 (2.09–3.42)	1.49 (1.14–1.94)
45–54	2.48 (1.91–3.22)	1.47 (1.10–1.96)
55–64	2.00 (1.45–2.76)	1.60 (1.13–2.26)
>65	0.58 (0.27–1.25)	0.55 (0.23–1.28)

*Ratios determined by using generalized linear models using a logarithmic linking function and Poisson distribution with robust variances. †Adjusted for all the covariates shown in the table.

## Conclusions

We show that a rapid increase occurred in LGV diagnoses in England in 2019, particularly affecting MSM who are HIV-negative or with unknown HIV status, such that nearly 60% of all LGV diagnoses are now in this group. This trend represents a major shift in the epidemiology of LGV; infection was previously associated with MSM living with HIV (*2*). However, having LGV diagnosed continues to be associated with living with HIV, as well as having a previous STI diagnosis, residing in London, and being born in LAC or Europe outside the United Kingdom.

An earlier decline in LGV diagnoses (*9*) has been attributed to revisions to LGV testing guidelines that led to expanded testing (*5*,*12*). However, because no revisions have been made since 2015, changes to testing practice are unlikely to explain the recent increase, which is concurrent with increases in test positivity (*10*). Of note, use of HIV preexposure prophylaxis (PrEP) in England may have contributed to increased testing for *C. trachomatis* and subsequent detection of LGV. Increased access to HIV prevention, including PrEP, in HIV-negative MSM engaging in high-risk sexual activities may have facilitated the change in the epidemiology of LGV and led to the observed increase in incidence among this group (*13*); the association with previous STI diagnosis further supports this hypothesis. Further investigation will be needed to understand the impact of HIV prevention on transmission of bacterial STIs. However, the increasing proportion of LGV diagnoses among HIV-negative MSM during 2017–2019 is in line with reports from other countries in Europe (*6*,*8*,*14*,*15*).

The first limitation of this study is that unmeasured behavioral covariates (e.g., number of partners, PrEP use, drug use, group sex, and venue-based activities) were not available. Inclusion of behavioral covariates in routine STI surveillance in England is underway and will be examined in future iterations of these analyses. Second, the risk among some groups, such as those not born in the United Kingdom, could be overestimated because of differing patterns of healthcare access and barriers to access.

In summary, we report a steep increase in the number of LGV diagnoses identified in SHS sites after a period of decline, which indicates the need for sustained surveillance and public health action. Our findings indicate that the epidemiology of LGV has changed, and an increased number of diagnoses are occurring among MSM who are HIV-negative or with unknown HIV status, highlighting the need to integrate health promotion and increase LGV testing within HIV prevention delivery. In addition, increased LGV risk among MSM born in LAC and in countries in Europe that are outside the United Kingdom indicates the need for increased accessibility of health promotion materials and wider engagement with these communities.
